# Dietary Intake and Risk for Reflux Esophagitis: A Case-Control Study

**DOI:** 10.1155/2013/691026

**Published:** 2013-04-16

**Authors:** Ping Wu, Xiao-Hu Zhao, Zi-Sheng Ai, Hui-Hui Sun, Ying Chen, Yuan-Xi Jiang, Yi-Li Tong, Shu-Chang Xu

**Affiliations:** ^1^Department of Clinical Nutrition, Tongji Hospital of Tongji University, Shanghai 200065, China; ^2^Department of Radiology, Tongji Hospital of Tongji University, Shanghai 200065, China; ^3^Department of Preventive Medicine, Tongji University School of Medicine, Shanghai 200092, China; ^4^Department of Gastroenterology, Tongji Hospital of Tongji University, Shanghai 200065, China

## Abstract

*Background*. Specific dietary components have been associated with gastroesophageal reflux disease (GERD) in Europe and the United States. However, the relationship between dietary components and GERD in Chinese still remains unclear. *Methods*. A total of 268 patients who were newly diagnosed as reflux esophagitis (RE) in Outpatient Endoscopy Center of Tongji Hospital were recruited. In addition, 269 sex- and age-matched subjects were also recruited as controls. The body measurements were determined, and the dietary intake during the previous year was evaluated using food frequency questionnaire (FFQ). Stepwise multiple logistic regression analysis was performed to examine the association between nutrients and RE. *Results*. After adjustment for WC, WHR, total energy intake, and demographics, there were a positive dose-response relationship between RE and calcium, meat, oils, and salt and a negative dose-response relationship between RE and protein, carbohydrate, calories from protein (%), vitamin C, grains and potatoes, fruits, and eggs. *Conclusion*. High intake of meat, oils, salt, and calcium is associated with an increased risk for RE while high intake of protein, carbohydrate, calories from protein (%), vitamin C, grains and potatoes, fruits, and eggs correlates with a reduced risk for RE.

## 1. Background

Gastroesophageal reflux disease (GERD) is a chronic disease usually caused by the reflux of acidic gastric and duodenal contents into the distal esophagus. The major symptoms of GERD include heartburn, acid regurgitation, and non-cardiac chest pain. GERD is a common digestive disease with the direct medical costs estimated around $9.3 billion annually [1], and with the symptoms portending a low quality of life [2]. Reflux esophagitis (RE) is one of the most common phenotypes of GERD [3]. In Western countries, GERD has a high prevalence. Especially in USA, about 44% of Americans suffer from GERD symptoms at least once monthly, 17% once weekly, and 7% once daily [4, 5]. Traditionally, GERD is less common in Asians [6]. However, it is reported that the prevalence of GERD in Asians is increasing [7]. The overall prevalence of RE in adult Japanese population is about 16% [8]. In Taiwan, the prevalence of RE is about 15% in patients evaluated for upper gastrointestinal tract symptoms [9] and about 10.5% in Korea healthy subjects [7]. In Chinese, few epidemiological data on GERD are available currently. In 1999, a Chinese study reported that the prevalence of GERD was 5.77% in Beijing and Shanghai, two biggest cities in China [10]. Although GERD is thought to be less prevalent in China than in Western countries and other Asian countries, recent studies reveal the incidence of GERD is on a rise in China [11].

Most of the factors involved in the pathogenesis of gastroesophageal reflux disease (GERD), previously described in European, Australian, and American studies, are present in Chinese patients with GERD, but at a lower scale. A low-fat diet probably contributes to a more favorable gastric distribution [12]. Another study reported GERD is highly prevalent in adult in Urumqi, especially in Uygur. Male, civil servant, smoking, strong tea, alcohol drinking, meat diet, and BMI are risk factors correlated to GERD [13]. In Europe and the United States, some investigators have shown that dietary fat, cholesterol, saturated fatty acid (SFA), dietary fiber, and other nutrients are associated with GERD. However, this association is absent in other studies. An epidemiologic survey showed that there was a link between high fat intake and GERD [14], and in clinical studies, esophageal pH provided direct evidence on the association between dietary fat and acid reflux. In contrast, a number of studies reported that a high-fat diet had no influence on the transient lower esophageal sphincter relaxation (TLESR) or esophageal acid exposure [15, 16]. Moreover, dietary fiber, especially cereal fiber, has been found to decrease the risk for esophageal and gastric adenocarcinoma [17], for which GERD is well-known risk factor. The mechanism may be that dietary fiber decreases the intake of gastric nitrites, which have been implicated in promoting reflux by relaxing the low esophageal sphincter (LES) [18]. Similar effects were also seen in a recent study by El-Serag et al. [14]. In this cross-sectional study, EL-serag and his colleagues postulated that high-fiber diet played a protective role. However, Bouin et al. [19] suggested that dietary fiber decreased the number of gastrooesophageal reflux, but increased their duration and had no significant effect on gastric emptying and gastric acid secretion. Another independent risk factor for GERD-related symptoms is alcohol [20], but some studies fail to identify such relationship [21, 22].

In general, the effects of diet on GERD are not well understood, and the currently available data in Western countries do not support a strong relationship between GERD and dietary fat, fiber, alcohol, and other nutrients. Although there are conflicting data regarding the role of dietary nutrients in GERD, there is no direct evidence that some nutrients promote or protect against GERD. Due to the difference in dietary nutrients between Chinese and Westerns, and few studies reporting the association between dietary nutrients and GERD in China, we employed food frequency questionnaire (FFQ) to evaluate the relationship between dietary components and RE in a Chinese population in the present study aiming to clarify whether the diet habits affect the prevalence of RE.

## 2. Materials and Methods

### 2.1. Patients

A total of 537 Han Chinese were recruited from the Endoscopy Center of Tongji Hospital between May 2010 and May 2011 in Shanghai. Because the diet habits vary in different peoples and Han Chinese account for 91.51% of population in China, the Han Chinese were recruited in order to maximally ensure the accuracy of data. Among these subjects, the age of 268 patients who were newly diagnosed as RE based on the Los Angles (LA) classification [23] ranged from 20 to 82 years, and 269 controls aged 19–80 years. The controls received routine health examinations including annual upper endoscopy, and all the controls were normal on upper gastrointestinal endoscopy and had no reflux symptoms. RE patients and controls were randomly selected and matched in the gender and age. Subjects were excluded if they had peptic ulcer (active or quiescent), endoscopic gastrointestinal tumors, history of upper gastrointestinal surgery, and over-the-counter medication (histamine-2-receptor antagonists, proton pump inhibitors, etc.) or were unable to complete the questionnaire and physical examination. 

### 2.2. Ethical Considerations

The whole protocol was approved by the Ethics Committee of Tongji Hospital. All subjects gave written informed consent before study. 

#### 2.2.1. Dietary Questionnaire

All subjects were trained to complete a detailed FFQ. Before survey, all subjects were required to complete a Reflux Diagnostic Questionnaire (RDQ), including “any symptoms including heartburn, acid regurgitation, and noncardiac chest pain,” and “often changing dietary habits and avoiding certain foods.” Controls with RDQ score of >12 were also excluded although the normal findings were present in the endoscopic examination. In order to avoid the influence of symptoms on the dietary intake, these subjects were asked to record the dietary intake before the onset of reflux symptoms. FFQ based on the Chinese Dietary Pagoda [24] was adapted for the Chinese population to enable completion within 40–50 min. A total of 120 kinds of food were included in the questionnaire based on the foods with high intake frequency in Chinese Nutrition Survey in 2002 and the new foods emerging in recent years. The food categories included grains, potatoes, meat, fish and shrimps, eggs, dark-colored vegetables, light-colored vegetables, fruits, nuts, beans and bean products, milk and dairy products, desserts, condiments, soft drinks, alcohol, western-style fast food, and animal oils. Participants were asked to report the foods (≥120) consumed in the past year. The intake of major foods was estimated according to the food moulds. The frequency of food intake in the FFQ ranged from “never or less than once monthly” to “twice daily.” Each question included three options for portion size. Using these data, the total frequency of intake of each food was calculated in a fixed period. The intake of each nutrient was calculated using the following formula: (reported intake frequency daily) × (portion size in grams) × (nutrient content per 100 grams)/100. The intake of plant oils, salt, and sugar was surveyed and converted according to the monthly consumption in each family and the number of family members.

#### 2.2.2. Anthropometric Measurements

The height, weight, waist circumference (WC), and hip circumference (HC) were measured under fasting conditions followed by endoscopy. Height was measured to the nearest 0.5 cm using a stadiometer, and weight to the nearest 0.25 kg in light clothing and without shoes using standard digital scales. BMI (kg/m^2^) was calculated as a ratio of weight (kg) to the square of height (m^2^). WC and HC were measured to the nearest 0.1 cm and the mean of three measurements was obtained. Waist-hip ratio (WHR) was calculated as a ratio of WC (cm) to HC (cm).

### 2.3. Quality Control and Methods

All investigators received professional training to collect and analyze data with stringent quality control standards. The investigators who collected anthropometric and dietary data were blind to the findings in endoscopy. An investigator supervised and checked all data. A nutrient calculator software designed by the Department of Clinical Nutrition of Tongji Hospital on the basis of China Food Composition Tables [25] was used to calculate the daily intakes of calories and nutrients.

### 2.4. Statistical Analysis

Statistical analysis was performed using SPSS version 14.0 for Windows (Chicago, IL, USA). All data were expressed as mean ± standard deviation (SD). *χ* ^2^ test and Kruskal-Wallis *H* test were used to compare the categorical variables, and *t* test to compare the parametric continuous variables. Stepwise multiple logistic regression analysis was employed to examine variables. The main predictors in the model were the dietary variables serving as continuous variables. The model was adjusted for the frequency matched variables: WC, WHR, total energy per day, age, sex, and education level. Odds ratios (OR) were calculated on the basis of interquartile range for each nutrient and thus show risk comparing the 75th centile of intake for each nutrient with the 25th centile. A value of *P* < 0.05 was considered statistically significant.

## 3. Results

### 3.1. Characteristics of Participants

Table 1 provides detailed characteristics of 537 subjects. RE patients were different from the controls in terms of education level (*P* < 0.05). RE patients had a higher WC and WHR than controls (*P* < 0.05) and there were no differences in the height, weight, BMI, and HC (*P* > 0.05) between them.  Table 2 displays the anthropometric measurements of two groups. The extent of oesophageal mucosal damage was assessed using the LA grading system [23]. Of the 268 patients with RE, 213 had grade A, 45 had grade B, 9 had grade C, and 1 had grade D oesophageal mucosal damage. Patients with mild RE accounted for 96.3% (Grade A and B).

### 3.2. Mean Daily Intake of Nutrients and Food

Data on nutrient and food intake obtained from the FFQ are shown in Table 3.

The daily intake of total energy, protein, fat, carbohydrate, total SFA, dietary fiber, selenium, milk and dairy products, beans, and nuts was significantly higher in the RE group than in the control group (*P* < 0.05). The calories from protein (%), calcium, *β*-carotene, vitamin C, and vegetables were markedly lower in the RE group than in the control group (*P* < 0.05).

There were no significant differences in the intake of calories from fats and carbohydrates (%), cholesterol, zinc, ferrum, vitamin E, grains and potatoes, fruits, meat, fish and shrimps, eggs, alcohol, oils, and salt (*P* > 0.05).

### 3.3. Relationship between RE and Intake of Various Nutrients and Food

After adjustment for WC, WHR, total energy intake, and demographics (sex, age and education level), there was a positive dose-response relationship between RE and calcium (OR 1.63, 95% CI 1.26–2.11), meat (OR 1.39, 95% CI 1.07–1.79), oils (OR 1.56, 95% CI 1.18–2.06), and salt (OR 9.93, 95% CI 5.33–18.49), and there was an inverse dose-response relationship between RE and protein (OR 0.68, 95% CI 0.47–0.98), carbohydrate (OR 0.66, 95% CI 0.45–0.97), calories from protein (%) (OR 0.64, 95% CI 0.48–0.84), vitamin C (OR 0.51, 95% CI 0.39–0.66), grains and potatoes (OR 0.58, 95% CI 0.39–0.85), fruits (OR 0.65, 95% CI 0.51–0.83), and eggs (OR 0.69, 95% CI 0.53–0.91).

After adjustment for WC, WHR, total energy intake, and demographics (sex, age, and education level), there was no correlation of RE with fat, total SFA, alcohol, cholesterol, calories from fat (%), calories from carbohydrate (%), dietary fiber, vitamin E, selenium, ferrum, zinc, *β*-carotene, vegetables, fish and shrimps, milk and dairy products, soy, and nuts. The relationship between RE and different nutrients and food is shown in Table 4.

## 4. Discussion

This is the first study reporting an association between the risk for RE and dietary nutrients as well as food in a Chinese population. In this study, results showed that the RE was mild (Grade A and B) which was similar to previously reported [26], and RE patients had higher WC and WHR when compared with healthy controls. Several previous studies have shown that overweight and obesity (especially abdominal obesity) are important independent risk factors for RE [27–32]. However, in Western countries, studies reveal that increased fat consumption (especially cholesterol and SFA rather than just weight disorder) has a dose-dependent correlation with GERD symptoms [14, 33, 34]. Therefore, experts in Western countries believe that food consumption patterns may be associated with the increasing prevalence of GERD, with low-fat and high-fiber foods playing a protective role, and high-vitamin C foods reducing the risk for GERD [35, 36]. In some clinical studies, esophageal pH provides direct evidence on the association between dietary fat and acid reflux. Shapiro et al. [33] found that, of all the dietary ingredients, cholesterol was the most important risk factor for intraesophageal acid reflux episodes in patients with GERD. It has been established that high-fat or large meal decreases the lower esophageal sphincter pressure (LESP), increases the rate of TLESR, and delays the gastric emptying [37], which may lead to a greater incidence of reflux [38]. Thus, it would be expected to increase the esophageal acid exposure in GERD [39]. Moreover, the dietary fiber has been found to decrease the risk for GERD, which may be attributed to the LES relax by dietary fiber. El-Serag et al. [14] recently reported that the daily intake of total fat, saturated fat, cholesterol, energy from dietary fat, and average fat servings in patients with GERD symptoms increased significantly when compared with subjects without GERD symptoms, and intake of high-fiber food correlated with a reduced risk for GERD symptoms. However, our results failed to establish the significant relationship between RE and fat as well as dietary fiber after adjustment for WC, WHR, total energy intake and demographics. Our results provided evidence supporting a link between the high consumption of meat and oils and the increased risk for RE, and the consumption of protein, carbohydrate, calories from protein (%), vitamin C, grains and potatoes, fruits, and eggs was related to the prevention against RE.

Some studies have demonstrated that an increased prevalence of reflux symptoms in alcohol users, and alcohol is an independent risk factor for GERD-related symptoms, with alcohol consumption exacerbating GERD by increasing acid secretion through gastric stimulation, reducing LESP, increasing spontaneous LES relaxations, and impairing esophageal motility and gastric emptying [20, 40–42]. Modest alcohol intake has been shown to induce reflux symptoms and decrease the esophageal pH in healthy individuals without GERD symptoms despite the overall 24 h pH was normal. Wang et al. [43] reported reflux symptoms in 43% of heavy (210 g/wk) alcoholics when compared with 16% of nondrinkers. In our study, no dose-response relationship was observed between RE and alcohol after adjustment for confounding variables, which was consistent with previously reported [21, 22]. The discrepancy may be due to the difference in the methodology. Pehl 1et al. showed that alcohol intake was correlated with obesity and suggested that patients with reflux symptoms should avoid intake of >300 mL of alcohol or beer [44].

Some previous studies found that an increase in salt consumption was associated with GERD [22, 45], which was attributed to the delayed gastric emptying and increased pancreaticobiliary secretion after high salt intake [46]. Similarly, our results also revealed a relationship between salt intake and RE. However, Aanen et al. [47] found that high dietary sodium did not increase the gastroesophageal reflux in healthy volunteers but reduced the LESP. Mizuta et al. also showed that slight increase in daily salt intake might be insufficient to affect the prevalence of RE [48]. Further investigations are needed to clarify this association.

Some experts proposed that high intake of vitamin C could exert a protective effect against GERD [35, 36]. Our findings revealed that excessive intake of animal products and less intake of vegetables may lead to vitamin C deficiency. Our results provided evidence supporting a relationship of high intake of vitamin C and fruits with prevention against RE. Our study suggests that RE patients should eat less energy-rich foods and more healthy foods such as vegetables and fruits, for health concerns.

Calcium is an important nutrient related to many diseases. However, to date, no studies have confirmed the relationship between calcium and GERD. To our knowledge, this is the first study to reveal the relationship between calcium intake and RE after adjustment for confounding variables. Nevertheless, the mechanism of such relationship is unknown. We speculated that calcium may stimulate the gastric acid secretion [49, 50], which may increase the esophageal acid exposure in GERD. Further studies are required to confirm the relationship between calcium and GERD.

The incidence of GERD is on the rise in China due to high intake of meat, oils, salt, and calcium, while high intake of protein, carbohydrate, calories from protein (%), vitamin C, grains and potatoes, fruits, and eggs correlates with a reduced risk for GERD, which is different from the findings in the study of El-Serag et al. The conflicting data may be attributed to differences in not only the race, geographic specificities, diet habit, and culture between Chinese and the Western, but the definition of GERD because studies based on GERD symptoms may be overinclusive, and our study based on GERD complications such as esophagitis is restrictive. Further studies are needed to clarify this association. 

Our study has some limitations: first, in the present study, incomplete data on vitamin and calcium supplements were not included for analysis, which may affect the determination of vitamin and calcium intakes; second, the folate, lutein, and other micronutrients were not employed for analysis and discussion since they are not included in Chinese Food Composition Tables; third, FFQ is not a particularly accurate dietary assessment tool, and there is potential for measurement error. However, FFQ is one of the most well validated and commonly used food frequency questionnaires; forth, the recall bias and residual confounding might also influence the results.

## 5. Conclusions

Our results indicate that high intake of calcium, meat, oils, and salt is associated with an increased risk for RE while high intake of protein, carbohydrate, calories from protein (%), vitamin C, grains and potatoes, fruits, and eggs correlates with a reduced risk for RE in Han Chinese. Further studies are required to explore the relationships among diet, obesity, and RE comprehensively.

## Figures and Tables

**Table 1:** Comparison of the RE group and control group.

Variables	RE group (%)	Control group (%)	*P *
	(*n* = 268)	(*n* = 269)	
Age (years)	50.9 ± 0.9	48.5 ± 0.8	0.055
mean (SD)			
20–29	21 (7.8)	27 (10.0)	0.181
30–39	41 (15.3)	47 (17.5)	
40–49	54 (20.2)	68 (25.3)	
50–59	81 (30.2)	60 (22.3)	
≥60	71 (26.5)	67 (24.9)	
Sex			
Men	137 (51.1)	131 (48.7)	0.575
Women	131 (48.9)	138 (51.3)	
Education level			
Illiteracy	21 (7.8)	16 (6.0)	*<*0.001
Primary school graduate	43 (16.0)	27 (10.0)	
Junior high school	79 (29.5)	103 (38.3)	
High school graduate	74 (27.6)	73 (27.1)	
College graduate	21 (7.8)	14 (5.2)	
Undergraduate	25 (9.3)	32 (11.9)	
Master graduate	5 (2)	4 (1.5)	

Abbreviations: RE: reflux esophagitis.

**Table 2:** Anthropometric measurements of RE group and control group.

Variables	RE group (%)	Control group (%)	*P *
	(*n* = 268)	(*n* = 269)	
Height	164.9 ± 0.5	164.4 ± 0.5	0.422
Weight	62.6 ± 0.7	62.8 ± 0.7	0.783
BMI	23.0 ± 0.2	23.2 ± 0.2	0.350
WC	82.8 ± 0.6	81.0 ± 0.6	0.031
HC	94.8 ± 0.4	94.2 ± 0.4	0.279
WHR	0.87 ± 0.0	0.86 ± 0.0	0.020

Abbreviations: RE: reflux esophagitis; BMI: body mass index; WC: waist circumference; HC: hip circumference; WHR: waist-hip ratio.

**Table 3:** Mean daily intake of nutrients and food in two groups.

Variables/day	RE group (*n* = 268)	Control group (*n* = 269)	*P *
Total energy (kcal)	2438.6 ± 53.7	2148.2 ± 38.4	<0.001
Macronutrients			
Protein (g)	78.2 ± 1.9	72.5 ± 1.4	0.019
Fat (g)	88.0 ± 3.2	71.6 ± 1.6	<0.001
Carbohydrate (g)	327.0 ± 7.6	297.7 ± 6.2	0.003
Total SFA (g)	21.4 ± 0.3	20.6 ± 0.2	0.035
Cholesterol (mg)	309.0 ± 10.0	297.5 ± 8.3	0.375
Calories from protein (%)	12.9 ± 0.2	13.7 ± 0.2	<0.001
Calories from fat (%)	31.6 ± 0.5	30.6 ± 0.4	0.103
Calories from carbohydrate (%)	54.8 ± 0.5	55.0 ± 0.5	0.705
Dietary fiber (g)	9.0 ± 0.3	8.2 ± 0.2	0.024
Micronutrients			
Zinc (mg)	11.9 ± 0.3	11.5 ± 0.2	0.281
Ferrum (mg)	17.6 ± 0.3	17.3 ± 0.3	0.467
Calcium (mg)	376.2 ± 7.8	426.1 ± 8.3	*<*0.001
Selenium (*μ*g)	52.9 ± 1.6	47.8 ± 1.1	0.010
*β*-carotene (*μ*g)	3322.2 ± 75	3676.2 ± 95.4	0.004
Vitamin E (mg)	50.6 ± 0.7	49.0 ± 0.6	0.076
Vitamin C (mg)	108.5 ± 4.5	136.0 ± 2.6	*<*0.001
Food			
Grains and potatoes (g)	371.6 ± 9.6	355.4 ± 8.3	0.202
Fruits (g)	102.8 ± 5.9	103.6 ± 5.6	0.924
Vegetables (g)	276.8 ± 6.0	344.6 ± 12.0	*<*0.001
Meat (g)	114.0 ± 7.9	102.4 ± 5.0	0.212
Fish and shrimps (g)	47.9 ± 2.9	49.0 ± 3.4	0.805
Eggs (g)	26.1 ± 1.3	26.5 ± 1.1	0.815
Milk and dairy products (g)	86.0 ± 5.6	68.7 ± 5.7	0.031
Beans and nuts (g)	16.1 ± 2.5	9.3 ± 1.0	0.011
Alcohol (g)	12.5 ± 2.3	8.0 ± 1.8	0.122
Oils (g)	42.3 ± 0.7	44.6 ± 2.0	0.263
Salt (g)	10.9 ± 0.1	10.9 ± 0.2	0.863

Abbreviations: RE: reflux esophagitis; SFA: saturated fatty acid.

**Table 4:** Risk for RE in patients with different intake of dietary nutrients and food groups.

Daily intake	OR	95% CI	*P *
Nutrients			

Protein (g/day)			
Unadjusted	1.01	0.79–1.28	0.97
Adjusted*	0.68	0.47–0.98	0.04
Fat (g/day)			
Unadjusted	1.32	1.04–1.68	0.02
Adjusted	1.24	0.93–1.85	0.14
Carbohydrate (g/day)			
Unadjusted	1.03	0.81–1.31	0.80
Adjusted	0.66	0.45–0.97	0.04
Total SFA (g/day)			
Unadjusted	1.06	0.83–1.35	0.65
Adjusted	1.05	0.81–1.37	0.70
Alcohol (g/day)			
Unadjusted	0.76	0.50–1.16	0.21
Adjusted	0.78	0.50–1.20	0.25
Cholesterol (mg/day)			
Unadjusted	1.05	0.83–1.34	0.67
Adjusted	0.96	0.74–1.25	0.78
Calories from protein (%)			
Unadjusted	0.64	0.50–0.81	*<*0.01
Adjusted	0.64	0.48–0.84	*<*0.01
Calories from fat (%)			
Unadjusted	0.94	0.74–1.19	0.58
Adjusted	1.04	0.79–1.37	0.80
Calories from carbohydrate (%)			
Unadjusted	0.95	0.74–1.20	0.64
Adjusted	0.95	0.74–1.23	0.70
Dietary fiber (g/day)			
Unadjusted	0.98	0.77–1.25	0.88
Adjusted	0.818	0.619–1.080	0.16
Vitamin C (mg/day)			
Unadjusted	0.52	0.40–0.66	*<*0.01
Adjusted	0.51	0.39–0.66	<0.01
Vitamin E (mg/day)			
Unadjusted	0.97	0.76–1.24	0.82
Adjusted	0.91	0.70–1.17	0.45
Selenium (*μ*g/day)			
Unadjusted	1.01	0.82–1.25	0.90
Adjusted	1.04	0.84–1.30	0.70
Ferrum (mg/day)			
Unadjusted	0.96	0.75–1.22	0.71
Adjusted	0.94	0.73–1.20	0.59
Zinc (mg/day)			
Unadjusted	0.99	0.78–1.25	0.91
Adjusted	0.96	0.75–1.23	0.75
Calcium (mg/day)			
Unadjusted	1.63	1.27–2.09	*<*0.01
Adjusted	1.63	1.26–2.11	<0.01
Beta-carotene (mg/day)			
Unadjusted	1.28	1.01–1.63	0.04
Adjusted	1.29	0.99–1.67	0.06

Food			

Grains and potatoes (g/day)			
Unadjusted	1.04	0.82–1.32	0.76
Adjusted	0.58	0.39–0.85	0.01
Fruits (g/day)			
Unadjusted	0.64	0.51–0.80	*<*0.01
Adjusted	0.65	0.51–0.83	<0.01
Vegetables (g/day)			
Unadjusted	1.09	0.78–1.51	0.62
Adjusted	1.13	0.80–1.58	0.49
Meat (g/day)			
Unadjusted	1.35	1.06–1.73	0.02
Adjusted	1.39	1.07–1.79	0.01
Fish and shrimps (g/day)			
Unadjusted	0.97	0.74–1.29	0.85
Adjusted	0.96	0.71–1.30	0.77
Eggs (g/day)			
Unadjusted	0.73	0.57–0.94	0.02
Adjusted	0.69	0.53–0.91	0.01
Milk and dairy products (g/day)			
Unadjusted	1.34	1.09–1.65	0.01
Adjusted	1.20	1.00–1.44	0.06
Soy and nuts (g/day)			
Unadjusted	1.17	0.95–1.44	0.15
Adjusted	1.09	0.92–1.31	0.33
Oils (g/day)			
Unadjusted	1.65	1.26–2.15	*<*0.01
Adjusted	1.56	1.18–2.06	<0.01
Salt (g/day)			
Unadjusted	9.10	5.18–16.00	*<*0.01
Adjusted	9.93	5.33–18.49	<0.01

Data are expressed as odds ratio with 95% confidence intervals (95% CI).

*Adjusted for WC, WHR, energy, age, sex, and education level.

Abbreviations: OR: odds ratios; CI: confidence interval; WC: waist circumference; WHR: waist-hip ratio.
